# Cytological structures and physiological and biochemical characteristics of covered oat (*Avena sativa* L.) and naked oat (*Avena nuda* L.) seeds during high-temperature artificial aging

**DOI:** 10.1186/s12870-024-05221-2

**Published:** 2024-06-11

**Authors:** Ruirui Yao, Huan Liu, Jinglong Wang, Shangli Shi, Guiqin Zhao, Xiangrui Zhou

**Affiliations:** 1 Key Laboratory of Grassland Ecosystems, College of Grassland Science, Gansu Agricultural University, Lanzhou, 730070 China; 2 Tibet Grassland Science Research Institute, Tibet Academy of Agricultural and Animal Husbandry Sciences, Lhasa, 850000 China

**Keywords:** Anatomy, Artificial aging, Biochemistry, Physiology, Ultrastructure

## Abstract

**Background:**

Seed aging, a natural and inevitable process occurring during storage. Oats, an annual herb belonging to the Gramineae family and pooideae. In addition to being a healthy food, oats serve as ecological pastures, combating soil salinization and desertification. They also play a role in promoting grassland agriculture and supplementing winter livestock feed. However, the high lipid and fat derivatives contents of oat seeds make them susceptible to deterioration, as fat derivatives are prone to rancidity, affecting oat seed production, storage, development, and germplasm resource utilization. Comparative studies on the effects of aging on physiology and cytological structure in covered and naked oat seeds are limited. Thus, our study aimed to determine the mechanism underlying seed deterioration in artificially aged ‘LongYan No. 3’ (*A. sativa*) and ‘BaiYan No. 2’ (*A. nuda*) seeds, providing a basis for the physiological evaluation of oat seed aging and serving as a reference for scientifically safe storage and efficient utilization of oats.

**Results:**

In both oat varieties, superoxide dismutase and catalase activities in seeds showed increasing and decreasing trends, respectively. Variance analysis revealed significant differences and interaction in all measured indicators of oat seeds between the two varieties at different aging times. ‘LongYan No. 3’ seeds, aged for 24–96 h, exhibited a germination rate of < 30%, Conductivity, malondialdehyde, soluble sugar, and soluble protein levels increased more significantly than the ‘BaiYan No. 2’. With prolonged aging leading to cell membrane degradation, reactive oxygen species accumulation, disrupted antioxidant enzyme system, evident embryo cell swelling, and disordered cell arrangement, blocking the nutrient supply route. Simultaneously, severely concentrated chromatin in the nucleus, damaged mitochondrial structure, and impaired energy metabolism were noted, resulting in the loss of ‘LongYan No. 3’ seed vitality and value. Conversely, ‘BaiYan No. 2’ seeds showed a germination rate of 73.33% after 96 h of aging, consistently higher antioxidant enzyme activity during aging, normal embryonic cell shape, and existence of the endoplasmic reticulum.

**Conclusions:**

ROS accumulation and antioxidant enzyme system damage in aged oat seeds, nuclear chromatin condensation, mitochondrial structure damage, nucleic acid metabolism and respiration weakened, oat seed vigor decreased. ‘LongYan No. 3’ seeds were more severely damaged under artificial aging than ‘BaiYan No. 2’ seeds, highlighting their heightened susceptibility to aging effects.

## Introduction

Oats, an annual herb belonging to the Gramineae family and pooideae [1], are the sixth most important cereal crop cultivated worldwide and are known for their resistance to soil infertility, salinity, and drought and cold conditions [2]. There are about 30 known oats species in the genus *Avena* in the world, covered oats (*Avena sativa* L.), an important forage crop, and naked oats (*Avena nuda* L.), which are crucial for green nutrition and commonly referred to as bell wheat. They are the two major species are mainly used in China. The difference between them lies in the presence or absence of lemma husks after threshing of the mature seeds, *Avena sativa* L. has hardened lemma husks and caryopsis, and the membranous lemmas of *Avena nuda* L. being separated from the caryopsis. Globally, 74% of oats contribute to livestock feed through seeds, leaves, and stalks [3]. In addition to being a healthy food, oats serve as ecological pastures, combating soil salinization and desertification. They also play a role in promoting grassland agriculture and supplementing winter livestock feed [4].

Germplasm resources are key to the development of the modern seed industry and form the basis for agricultural science and technology innovation [2]. Successful agricultural production depends on the availability of high-quality seeds [5]. Preserving seed quality and vigor is crucial for food consumption and seed storage. Although oat seeds maintain high internal vigor for a short period, prolonged storage results in irreversible deterioration, known as aging. Aged seeds lose viability and show low germination potential (GP), germination rate (GR), germination index (GI) and vigor index (VI) and increased sensitivity to stress upon germination. In addition, seeds deterioration manifests as irreversible metabolic and cellular changes, such as reduced antioxidant capacity, plasma membrane damage, depletion of nutrient storage and destruction of genetic materials [6]. When subjected to stress such as aging, plants will initiate endogenous hormone regulation programs to resist stress and control their own growth and development. The relative proportion of growth-promoting hormones (such as Gibberellin (GA_3_), Indoleacetic acid (IAA) and Cytokinins (CTK)) and growth-inhibiting hormones (Abscisic Acid (ABA)) in plant seeds is one of the main factors that determine whether seeds can germinate [7]. Oat grains have a well-balanced distribution of proteins, soluble dietary fiber, β-glucan, unsaturated fatty acids, vitamins, and minerals essential for human health [8, 9]. However, the high lipid and fat derivatives contents of oat seeds make them susceptible to deterioration, as fat derivatives are prone to rancidity, affecting oat seed production, storage, development, and germplasm resource utilization [10–13].

Seed aging, a natural and inevitable process occurring during storage [14], proceeds slowly under normal storage conditions. However, the aging process can be accelerated by exposing seeds to high temperatures and humidity, with researchers using this technique for artificial aging as a substitute for natural aging [15]. Fu et al. analyzed the cellular morphology of naturally and artificially aged flax seeds, using terminal deoxynucleotidyl transferase-mediated dUTP nick end labeling and an assay coupled with 4,6-diamidino-2-phenylindole staining [15]. Similarly, Rajjou et al. reported increased protein oxidation (carbonylation), loss of functional properties in seed proteins and enzymes, and enhanced susceptibility to protein hydrolysis in *Arabidopsis thaliana* L. seeds during both artificial and natural aging [16]. Nonetheless, significant differences between artificial aging and natural aging, controlled by different genes, have been identified [17, 18]. Unique *QTLs* (*qSSnj-2-1* and *qSSn-2-2*) were detected only after artificial aging, indicating that artificial treatments incompletely replicate the deterioration processes observed under conventional storage conditions [19]. Both aging processes induce changes in biochemical reactions and physiological seed structure [15]; however, further study is necessary to clarify whether the aging mechanisms are identical. Research on artificially aged oat seeds has predominantly focused on seed initiation, germination characteristics, and genetic integrity [2, 3, 20, 21]. Comparative studies on the effects of aging on physiology and cytological structure in covered and naked oat seeds are limited. The present study explored seed viability, cellular anatomical structure, seed embryos, and mitochondrial ultrastructure in two oat germplasm varieties. This study aimed to determine the mechanism underlying seed deterioration in artificially aged covered and naked oats, providing a basis for the physiological evaluation of oat seed aging and serving as a reference for scientifically safe storage and efficient utilization of oats.

## Results

### Effects of different aging times on the seed vigor of covered and naked oats

**Fig. 1:**
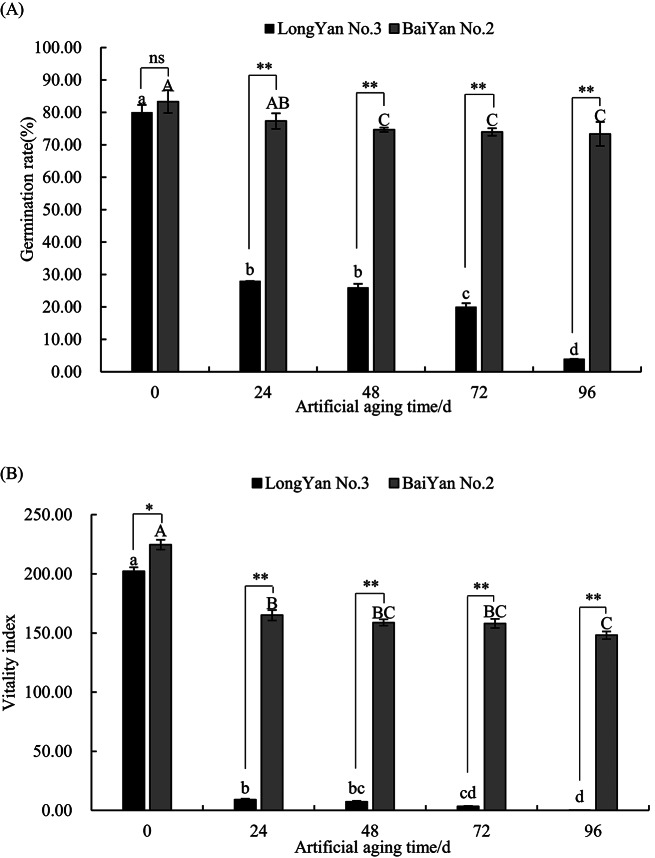
Effect of artificial aging treatments on the seed viability of covered and naked oat seeds. (**A**): Germination rate of oat seeds. (**B**): Vigor index of oat seeds. Data are presented as mean ± SD (*n* = 3 biological replicates, each with 50 seeds). The uppercase and lowercase letters indicate significant differences (*P* < 0.05) at various aging treatment times for the same oat variety. The symbols ns, *, and ** indicate nonsignificant (*P* > 0.05), significant (*P* < 0.05), and highly significant (*P* < 0.01) differences, respectively, between the two oat varieties at the same aging time, the same as below

The germination rate and vigor index of ‘LongYan No. 3’ and ‘BaiYan No. 2’ seeds decreased with intensified artificial aging. Significant and highly significant differences (*P* < 0.05 and *P* < 0.01, respectively) were observed in the seed germination percentage and vigor index of the two varieties under aging treatments lasting 0–96 h. After 24 h of seed aging, the germination rate and vigor index of ‘LongYan No. 3’ seeds significantly decreased by 65% and 95.46%, respectively, compared with those of unaged seeds (*P* < 0.01). After 24 h of seed aging, the vigor index of ‘BaiYan No. 2’ decreased by 26.55% compared with that of unaged seeds (*P* < 0.05), whereas no significant difference in germination rate was observed. During 48–96 h of aging, the germination rate and vigor index of ‘BaiYan No. 2’ seeds decreased slightly but nonsignificantly (*P* > 0.05), with both values remaining relatively high. In contrast, at 96 h of aging, the seed germination rate and vigor index of ‘LongYan No. 3’ decreased to their lowest levels: 4% and 0.07%, respectively (Fig. 1A, B).

**Fig. 2:**
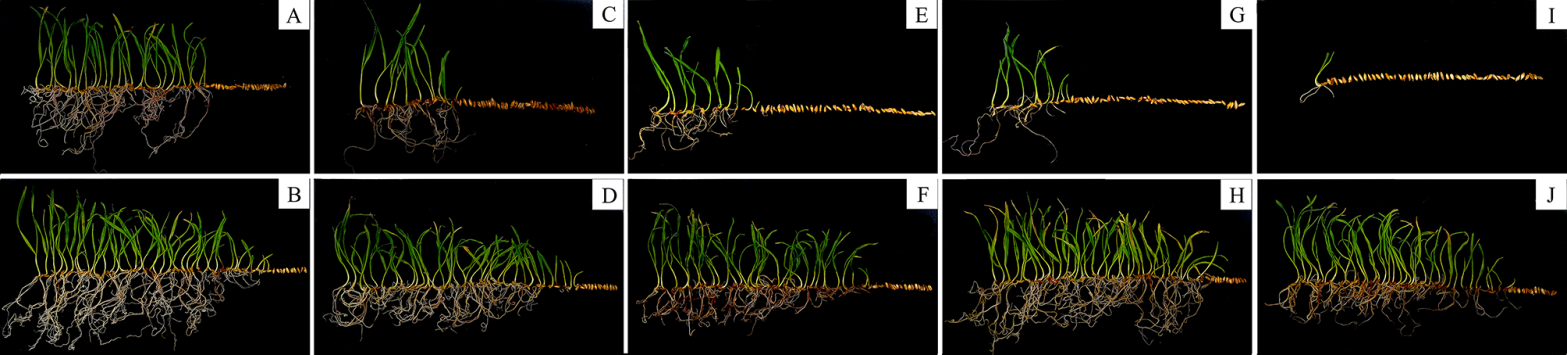
Effect of aging time on seed viability in covered and naked oats. **A, C, E, G, I**: Artificial aging for 0, 24, 48, 72, and 96 h in ‘LongYan No. 3’ oat seeds. **B, D, F, H, J**: Artificial aging for 0, 24, 48, 72, and 96 h in ‘BaiYan No. 2’ oat seeds

As the artificial aging duration increased, ‘LongYan No. 3’ seed viability decreased, reaching its lowest level at 96 h. Conversely, the seed viability of ‘BaiYan No. 2’ remained high and largely unchanged, even after 96 h of aging (Fig. 2).

Significant differences (*P* < 0.01) in the germination percentage and vigor index of oat seeds were observed between the two varieties at different aging times. The interaction of variety and aging time showed highly significant differences (*P* < 0.01) in germination percentage and vigor index, indicating that aging time markedly affected the vigor of both oat seed varieties (Table 1).

**Table 1:** Analysis of variance of aging treatment time and seed germination in covered oats and naked oats

Index	Germination rate	Vitality index
Variety	2655.39**	7194.00**
Artifical aging time	127.43**	857.25**
Variety×Artifical aging time	86.64**	369.24**

### Physiological and biochemical indexes of covered and naked oat seeds at different aging times

**Fig. 3:**
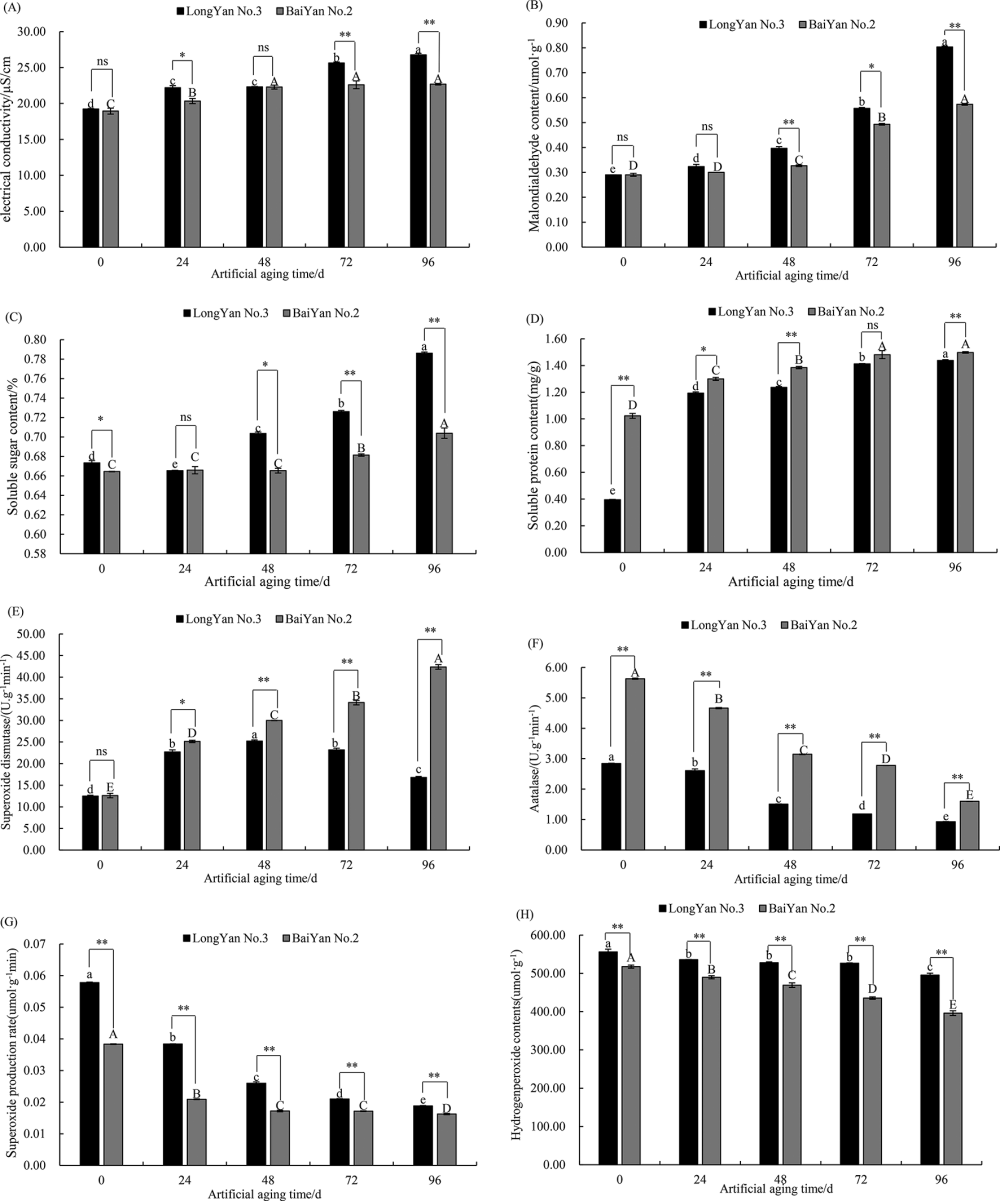
Effect of aging time on physiological and biochemical indexes of covered oats and naked oats. (**A**) the conductivity, (**B**) MDA, (**C**) SS, (**D**)SP, (**E**) SOD, (**F**) CAT, (**G**) O2^·-^and (**H**) H_2_O_2_ content during seed aging process

With prolonged artificial aging time, the conductivity of the seed leachate in both ‘LongYan No. 3’and ‘BaiYan No. 2’ varieties gradually increased. No significant difference (*P* > 0.05) was observed between the seed conductivities of the two varieties at the start of artificial aging (0 h); however, a highly significant difference (*P* < 0.01) was noted after 72 h of aging. Compared with the start of aging (0 h), the electrical conductivity of ‘LongYan No. 3’ and ‘BaiYan No. 2’ seeds increased by 13.33% (*P* < 0.05) and 6.85% (*P* < 0.05) after 24 h of aging and by 28.15% and 16.49% (*P* < 0.05) after 96 h of aging, respectively (Fig. 3–A).

Both oat germplasms showed a gradual increase in MDA content with prolonged aging time. Significant (*P* < 0.05) or highly significant (*P* < 0.01) differences in MDA content were observed between the two germplasms. ‘LongYan No. 3’ showed an increased degree of seed deterioration, whereas ‘BaiYan No. 2’ was less affected by the artificial aging process. At 96 h of aging, the MDA content of ‘LongYan No. 3’ and ‘BaiYan No. 2’ seeds reached peak values, which were 63.90% and 49.42% higher, respectively, than that of the control (Fig. 3–B).

The SS content of ‘LongYan No. 3’ and ‘BaiYan No. 2’ seeds exhibited a trend of slow and then rapid growth after 48–96 h of aging, with highly significant differences observed in the SS content of both seeds (*P* < 0.01). At 48 h of aging, the SS content of ‘LongYan No. 3’ and ‘BaiYan No. 2’ seeds increased by 4.29% (*P* < 0.05) and 0.15% (*P* > 0.05), respectively, compared with that at 0 h of aging. At 96 h of aging, the SS content of both varieties reached peak values, which were 14.33% (*P* < 0.05) and 5.61% (*P* < 0.05) higher, respectively, than that of the control (Fig. 3–C).

Throughout the aging process, the SP content of oat seeds exhibited an increasing trend, with highly significant differences observed in the SP content of the two varieties (*P* < 0.01). At 96 h of aging, the SP content of ‘LongYan No. 3’ and ‘BaiYan No. 2’ seeds reached peak values, which were 72.47% and 31.68% higher, respectively, than that at 0 h of aging (*P* < 0.05) (Fig. 3–D).

No significant difference (*P* > 0.05) was observed in superoxide dismutase (SOD) activity between unaged ‘LongYan No. 3’ and ‘BaiYan No. 2’ seeds. With prolonged aging time, ‘LongYan No. 3’ seeds showed an increasing and then decreasing trend in SOD activity, whereas ‘BaiYan No. 2’ seeds exhibited a continuous increase in SOD activity. SOD activity in the two germplasms differed significantly (*P* < 0.05 or *P* < 0.01) at 24–96 h of aging. The SOD activity of ‘LongYan No. 3’ and ‘BaiYan No. 2’ seeds reached peaked values at 48 and 96 h of aging, which were 50.46% and 70.27% higher, respectively, than that of control seeds (Fig. 3–E).

The CAT activity of the seeds gradually decreased with prolonged aging time, with significant differences (*P* < 0.01) observed in the CAT activities of the two germplasms. The CAT activity in ‘LongYan No. 3’ and ‘BaiYan No. 2’ seeds reached the lowest levels at 96 h of aging, showing significant decreases of 67.32% and 71.55%, respectively, compared with that of control seeds. Throughout the aging process, ‘BaiYan No. 2’ seeds had higher CAT activity than ‘LongYan No. 3’ seeds (Fig. 3–F).

The rate of O_2_·^−^ in oat seeds showed a decreasing trend throughout the aging process, with a highly significant difference observed between the two varieties (*P* < 0.01). The rate of O_2_·^−^ was the lowest at 96 h of aging, decreasing by 67.47% and 57.44% in ‘LongYan No. 3’ and ‘BaiYan No. 2’ seeds, respectively, compared with that in the control (*P* < 0.05) (Fig. 3–G). The H_2_O_2_ content of oat seeds gradually decreased with prolonged aging time (Fig. 3–H).

Significant differences (*P* < 0.01) were observed in oat seed SS content, SP content, seed SOD and CAT activities, and O_2_·^−^ and H_2_O_2_ content across varieties and aging times, with significant differences (*P* < 0.01) in the same variables observed under the interaction of variety and aging time. This indicates that the interaction effect exists, with aging time markedly influencing physiological changes in different oat varieties (Table 2).

**Table 2:** Analysis of variance of physiological and biochemical indexes of covered and naked oat seeds at different aging times

Index	EC	MDA content	SS content	SP content	SOD	CAT	O_2_^·−O^_2_^·−^ content	H_2_O_2_ content
Variety	15220.71**	22973.74**	203920.67**	23871.63**	11331.10**	64417.68**	57316.47**	32331.32**
Artifical aging time	125.43**	2697.42**	356.74**	1167.03**	677.47**	8047.86**	8821.84**	122.43**
Variety×Artifical aging time	45.24**	818.47**	156.88**	424.28**	440.46**	2612.19**	2831.38**	45.08**

### Effects of different aging times on the anatomical structure of covered and naked oat seeds

**Fig. 4:**
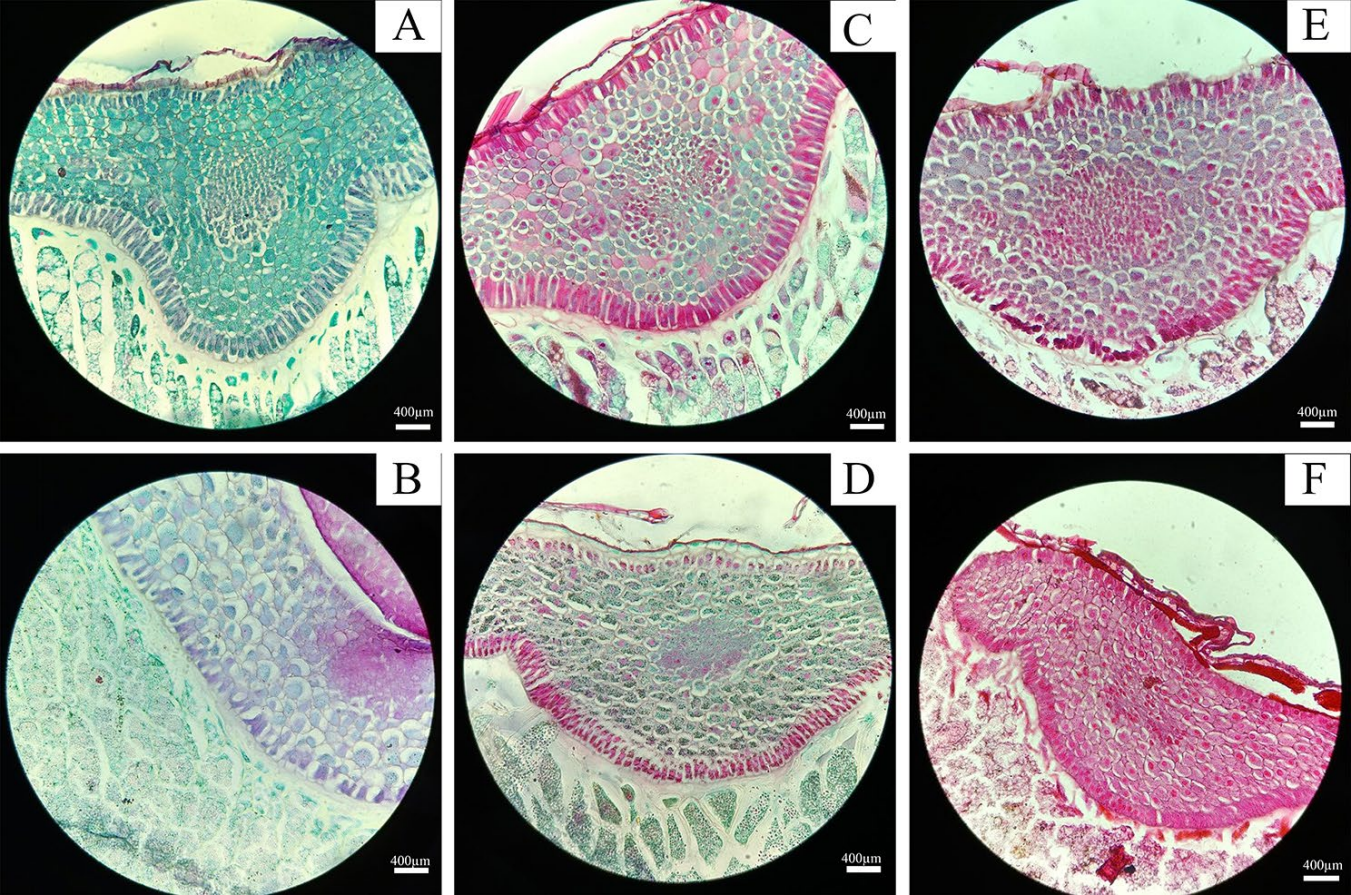
Effects of artificial aging time on the microstructures of covered and naked oat seeds. **A, C, E**: Microstructure of ‘LongYan No. 3’ seed embryos following artificial aging for 0, 24, and 96 h. **B, D, F**: Microstructure of ‘BaiYan No.2’ seed embryos following artificial aging for 0, 24, and 96 h

The microstructure of ‘LongYan No. 3’ and ‘BaiYan No. 2’ oat seed embryos changed with increasing artificial aging treatment time. Without artificial aging, the microstructure of the embryos of both varieties appeared normal. The cells in the embryo were lightly stained with Safranin O and Fast Green. closely arranged, and maintained their typical shape. The cells at the junction of the seed embryo and the endosperm were neatly and orderly arranged (Fig. 4–A, B). After 24 h of artificial aging, the cells in the embryo of ‘LongYan No. 3’ seeds were deepened by Safranin O and Fast Green staining, indicating swelling and plasmic wall separation. Additionally, cells at the junction of the seed embryo and the endosperm were stained reddish (Fig. 4–C). Conversely, no notable changes were observed in the cells of ‘BaiYan No. 2’ seed embryos. The cells were closely arranged in an orderly manner, with those at the junction of the embryo and the endosperm stained red by Safranin O and Fast Green staining (Fig. 4–D). After 96 h of artificial aging, the ‘LongYan No. 3’ seed embryo cells exhibited significant swelling. The cell gap increased, cell staining at the junction between the embryo and endosperm deepened, and the cell arrangement became disordered (Fig. 4–E). Similarly, cells in ‘BaiYan No. 2’ seed embryos were deepened by Safranin O and Fast Green staining after 96 h of artificial aging, with some cells slightly swollen and those at the junction between the seed embryo and the endosperm pink with a tight and orderly cell arrangement (Fig. 4–F).

### Impact of varying aging times on the ultrastructure of embryo cells and mitochondria in covered and naked oat seeds

**Fig. 5:**
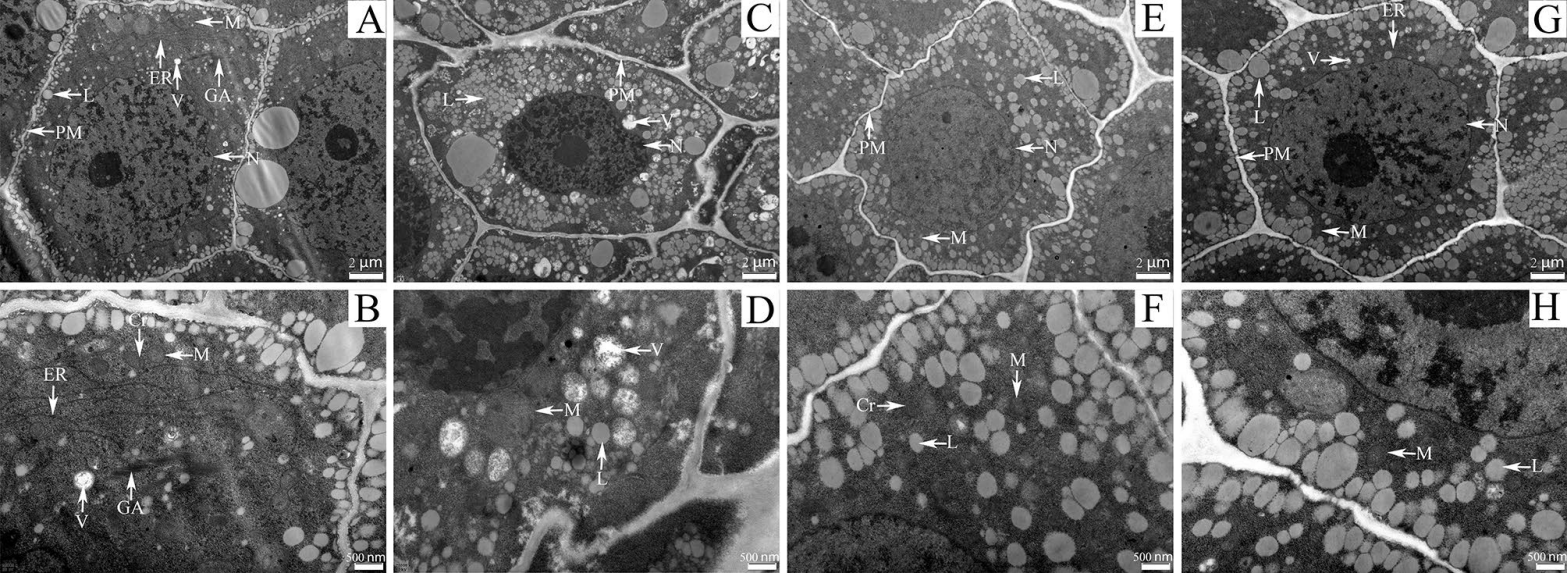
Ultrastructural changes in the seed embryos and mitochondria of covered and naked oats at different aging times. **A–D**: ‘LongYan No. 3’ variety; **A, B**: Oat seeds artificially aged for 0 h; **C, D**: Oat seeds artificially aged for 96 h; **E–H**: ‘BaiYan No. 2’ variety; **E, F**: Oat seeds artificially aged for 0 h; **G, H**: Oat seeds artificially aged for 96 h. Abbreviations: L: lipid droplet; M: mitochondria; N: nucleus; V: vacuolar structure; PM: cytoplasmic membrane; ER: endoplasmic reticulum; GA: Golgi apparatus; Cr: cristae

Electron microscopy images revealed the ultrastructure of oat seed embryos (Fig. 5). In both ‘LongYan No. 3’ and ‘BaiYan No. 2’ varieties, seed embryo cells maintained a normal state without artificial aging. The cell structure, organelles, nuclear membrane, and euchromatin distribution in the nucleus were all normal. The nucleus appeared lighter, and the nucleolus was dense with a clearly defined nucleoplasm, indicating appropriate structure and function. The mitochondria exhibited a flat or ellipsoid shape, with visible mitochondrial and nuclear membranes. Additionally, the mitochondrial matrix was dense, with ridges observed at a few sites. ‘LongYan No. 3’ seed embryo cells contained abundant Golgi apparatus and endoplasmic reticulum, with fat bodies concentrated around the cell membrane. In contrast, ‘BaiYan No. 2’ seed embryo cells were covered with numerous fat bodies (Fig. 5–A, B, E, F).

After 96 h of artificial aging, seed embryo cells of both oat varieties underwent several changes. In ‘LongYan No. 3’ embryos, the number of fat bodies significantly increased, with many fat bodies being dissolved and gradually engulfed by the original vesicles, forming large vesicles that filled the entire cell. The cell gap also increased significantly, and the chromatin in the nucleus showed severe condensation, leading to lysed nuclear membranes. The mitochondrial matrix was diluted, ridges were not observed, and the number of endoplasmic reticulum and Golgi apparatus decreased. In ‘BaiYan No. 2’ variety, the shape of embryo cells remained normal after 96 h of aging, with visible endoplasmic reticulum. The chromatin in the nucleus of embryo cells was slightly condensed, and some fat bodies appeared lysed. The nuclear membrane remained intact and clear, and some part of the mitochondrial matrix was diluted without observable ridges (Fig. 5–C, D, G, H).

## Discussion

### Effects of aging time on the seed viability and physiological and biochemical properties of covered and naked oat seeds

In practice, storage of seeds at low temperatures and low moisture levels is ideal for inhibiting seed deterioration [22]. Most previous studies have reported a decline in seed vigor after aging treatments [23, 24]. These treatments are essential for understanding the mechanisms underlying seed aging and detecting seed viability [16, 25]. In the current study, significant differences and interactions (*P* < 0.01) in seed germination and vigor index were noted between covered and naked oat varieties at different aging times. The germination rate and vigor index of ‘LongYan No. 3’ seeds decreased significantly faster than those of ‘BaiYan No. 2’ seeds during the aging process (*P* < 0.05). A previous study reported that the decline in vigor indexes of ‘BaiYan No. 2’ seeds was significantly faster than that of ‘LongYan No. 3’ seeds with prolonged storage [26]. This difference in the magnitude of change in seed vigor between natural and artificial aging was evident, with the aging rate of ‘LongYan No. 3’ also differing between the two aging processes. Under natural aging, ‘LongYan No. 3’ exhibited a slightly slower decline in seed vigor, likely due to the protective effect of its lemma and seed coat at natural storage temperatures. In contrast, artificial aging under high temperature and high humidity conditions resulted in breakage of the external structure, leading to a rapid decline in the seed vigor and aging rate of covered oat seeds. This highlighted their lower storage tolerance compared with that of naked oats. Additionally, ‘LongYan No. 3’ seed embryonic cells experienced significant swelling and plasmic wall separation during early aging stages. As aging progressed, cell swelling became more pronounced, the gap becomes larger and disorderly arrangement. In contrast, ‘BaiYan No. 2’ seed embryonic cells exhibited only slight swelling, maintaining a tight and orderly arrangement.

Seed aging starts with membrane damage, reflected in increased membrane permeability, resulting in the leakage of soluble nutrients and physiologically active substances, observed as elevated electrical conductivity in the osmotic fluid of seed cells [27, 28]. The viability of ‘LongYan No. 3’ and ‘BaiYan No. 2’ seeds decreased under aging treatment, evident in the gradual increase in conductivity and MDA content of the oat seed leachate. This suggests the occurrence of oxidative damage to membrane lipids, leading to degradation of the cell membrane and its semipermeable membrane function [29, 30]. Under normal physiological conditions, the production and removal of reactive oxygen species (ROS) in seeds are balanced. Under stress or aging, seeds experience an imbalance in the generation and removal of ROS, leading to ROS accumulation, which triggers or exacerbates lipid peroxidation, degrading unsaturated fatty acids in the biofilm, leading to membrane structure destruction and impaired function [31, 32]. In the present study, aging treatment significantly increased O_2_·^−^ production in oat seed cells. In the early stage of aging, SOD activity gradually increased while O_2_·^−^ production slowed down, indicating enhanced disproportionation of O_2_·^−^ and its conversion to H_2_O_2_ and O_2_ [33, 34]. Additionally, CAT, which scavenges H_2_O_2_ under oxidative stress, exhibited decreased activity as aging progressed, contributing to H_2_O_2_ and other ROS accumulation, thereby exacerbating membrane lipid peroxidation, damaging the oat seed’s antioxidant enzyme system, and reducing seed viability [35, 36]. ‘BaiYan No. 2’ seeds consistently exhibited higher antioxidant enzyme activity and greater ROS scavenging ability compared with ‘LongYan No. 3’ seeds. The decrease in ROS levels after reaching their peak is attributed to the antioxidant system’s scavenging effect and damage caused by ROS accumulation to the membrane and mitochondrial structure and function, as well as protein denaturation. This process resulted in an overall decrease in seed metabolism, leading to the gradual decrease in ROS content with seed death. Active oxygen has a great influence on seed vigor, so the production of active oxygen in seeds should be avoided during seed storage. Zinc has been proven effective in reducing ROS generation and defending cells against ROS attack, while Zn deficiency allows higher ROS accumulation causing plant damage [37]. The UV absorption efficiency of nano-ZnO (n-ZnO) makes them highly effective for improving antioxidant enzyme activities, growth and plant performance against abiotic stress [38, 39]. Therefore, in subsequent studies, it is desired to combine aging with n-ZnO pretreatment.

### Effect of aging time on the anatomical structures of covered and naked oat seeds

The success of cereal grain germination and initial stages of seedling growth depends on the precise organization of events taking place during this process. This study revealed that high temperature and high humidity conditions accelerated the deterioration of oat seeds. Changes in the moisture content within the seed, driven by environmental humidity, induce horizontal exchange between the seed and environment. The seed hygroscopicity amplifies respiration intensity and hydrolytic enzyme activity (such as protease activity), accelerating nutrient decomposition and seed deterioration [40]. Storage compounds, such as starch, protein, and fat, accumulate primarily in mature seeds in the endosperm and are ready to be mobilized during seed imbibition and subsequent germination [41]. These compounds are hydrolyzed into sugars, amino acids, and fatty acids by the action of amylase, protease, and esterase, respectively. Then, these small molecules produce sugar compounds through deamidation and β-oxidation. Finally, mainly in the seed embryo, sugar compounds through glycolysis and pentose phosphate pathway provide sufficient energy for seeds germination [42–44]. Anatomically, oat seeds are similar to the seeds of other graminaceous plants. Paraffin sections revealed that oat seeds consist of a seed coat, embryo, and endosperm, with the embryo concealed within the endosperm [45]. Under ambient storage conditions, these internal tissues form a cohesive structure, with tight binding between various tissues. Following artificial aging under high temperature and high humidity conditions, the embryo cells of ‘LongYan No. 3’ seeds exhibited swelling, with some cells separating from the plasma wall. As aging progressed, the swelling intensified, resulting in larger gaps between cells, leading to a disordered cell arrangement. This change may hinder the physiological pathway of gibberellin into aleurone layer, preventing starch hydrolysis enzymes from entering the endosperm, thereby blocking the nutrient supply pathway and causing a decline in ‘LongYan No. 3’ seed vitality [46]. The aleurone cells are able to perceive gibberellins and induce the synthesis and secretion of hydrolytic enzymes, these cells play a central role to mobilize the storage material of the starchy endosperm and have received more attention than any other grain tissue. Once the aleurone cells have performed their important function, enter in a process of Programmed Cell Death (PCD), which is also activated by gibberellins [47], thus allowing the use of the aleurone cellular contents for seedling growth. ABA is also reported to inhibit seed germination by inhibiting GA_3_ biosynthesis directly under high temperature [48]. ‘BaiYan No. 2’ seeds showed some swelling in embryonic cells but remained neatly arranged, indicating minimal impact of the aging process on naked oat seed viability. After 8 years of storage, the cells of the aleurone layer of ‘LongYan No. 3’ seeds showed slight fragmentation, whereas those of ‘BaiYan No. 2’ exhibited major breakage, enlarged cell gaps, and severely degraded protein storage vacuoles, with numerous droplet-like structures appearing inside the cells [26]. Scutellar cells undergoing PCD show vacuolization in the cytoplasm and a proactive intramembrane system linking the intracellular secretory pathway to a process of vacuolar cell death [49]. The presence of precursor protease vesicles and autolytic compartments derived from the endoplasmic reticulum [50, 51] and Golgi cisternae [52] are considered as features of plant cell death, resembling morphological features of autophagy in animal cells. Although the role of autophagy in cell death is still subject of discussion [53], both morphological and biochemical evidence suggests that autophagy has a pro-death function either in developmental [54] or pathogen-induced PCD in plants [55]. Under natural storage conditions, the external structures of covered oat protect the caryopsis from various biotic and abiotic stresses, with the lemma playing a protective role. However, the internal structure of ‘BaiYan No. 2’ (naked oat) seeds was affected by artificial aging under high temperature, confirming that this process compromises the external structure protective ability while also highlighting the lower storage resistance of covered oats compared with that of naked oats.

### Impact of various aging times on the ultrastructure of seed embryo cells and mitochondria in covered and naked oat seeds

Without artificial aging, normal oat seed vesicles displayed intact organelles and debris-free internal structures, with an intact vesicle membrane, electron-dense material along the membrane, and well-distributed mitochondria and plastids in the cytoplasm. Endoplasmic reticulum and Golgi apparatus structures remained intact, indicating the presence of normal cell metabolism. The euchromatin of oat seed embryo cells was evenly distributed in the nucleus, resulting in an overall lighter cell color. During this period, the DNA double helix structure in the nucleus mainly exists in a relaxed state, and nucleic acids undergo active transcription and synthesis metabolism. A characteristic feature of PCD is that mitochondria show varying degrees of structural alteration. After subjecting oat seeds to high temperature and high humidity conditions, the mitochondrial matrix was diluted and the inner cavity showed vacuolization. This indicates severe mitochondrial damage, resulting in impaired energy metabolism and cellular senescence. Wang et al. revealed that ultrastructural damage in wheat embryo cells became more severe as storage temperature and humidity increased [56]. After aging, the chromatin in the nucleus of oat seed embryo cells became concentrated and the nuclear membrane was dissolved, which indicated weakened nucleic acid metabolism, contributing to reduced seed viability. Cytological changes, particularly in the mitochondrial ultrastructure, occur as seeds deteriorate in response to aging-induced oxidative stress. The mitochondria serve as the primary organelle responsible for producing and being susceptible to free radicals, which act as the energy factory for cellular life activities [57, 58]. When plants are subjected to aging stress, damage to mitochondria, rupture of the mitochondrial membrane or damage to the mitochondrial COX respiratory pathway can result in a blockage of the formation of the transmembrane proton gradient, or an increase in AOX activity can lead to an increase in oxygen consumption in the respiratory electron transport chain but not a significant increase in the transmembrane proton gradient [59], and increased UCP activity leads to a decrease in the transmembrane proton gradient used to drive ATP synthesis [60, 61]. All of these reasons lead to uncoupling of electron transfer from oxidative phosphorylation, making mitochondrial oxidative phosphorylation less efficient. so that the efficiency of mitochondrial oxidative phosphorylation is reduced, respiration is weakened, so that the ATP supply is insufficient during oat seed germination, electron leakage occurs, and free radicals are prompted, leading to a decrease in seed vigor, a decrease in the rate of germination, and t affecting the morphology of the seedling establishment.

## Conclusion

With prolonged artificial aging, significant changes were observed in the anatomical structure of oat seeds and the ultrastructure of seed embryo cells and mitochondria. During the artificial aging process, seed embryo cells exhibited swelling and large cell gaps. The nuclear chromatin in aged oat seed embryo cells condensed, and the mitochondrial matrix became diluted and vacuolated, leading to weakened cell metabolism in the oat seed embryo. This weakening ultimately resulted in reduced seed viability, highlighting the close relationship between structure and function. The reduction of the cell membrane’s ability to act as a semipermeable membrane coupled with the accumulation of ROS in the seeds contribute to the destruction of the oat seed antioxidant enzyme system with prolonged aging. The swelling phenomenon in seed embryo cells became more pronounced, disrupting the cellular arrangement and blocking nutrient supply pathways. Additionally, chromatin condensation in the cell nucleus, mitochondrial structure breakage, and impaired normal cell energy metabolism further contribute to the decline in seed vigor, rendering ‘LongYan No. 3’ seeds nonviable. The germination rate and antioxidant enzyme activity of ‘BaiYan No. 2’ seeds were consistently higher than those of ‘LongYan No. 3’ seeds, and the shape of their seed embryo cells remained normal, with visible endoplasmic reticulum. Overall, the deterioration observed in artificially aged ‘LongYan No. 3’ seeds with husks was more pronounced than that in ‘BaiYan No. 2’ naked oats.

## Materials and methods

### Materials

The test materials were the ‘LongYan No. 3’ variety of covered oats (*Avena sativa* L.) and ‘BaiYan No. 2’ variety of naked oats (*Avena nuda* L.). ‘LongYan No. 3’ was a new oat variety bred by crossing Danish black-seed oat 444 as female parent and European black oat Fyris as male parent in 1998. It was selected by Gansu Agricultural University. ‘LongYan No. 3’ has been reported to have superior yield and high nutritional quality, which makes it suitable for cultivation in Tongwei County and similar high-humid mountain area in Gansu Province [62]. ‘BaiYan No. 2’ was bred by pedigree method based on the F4 generation of oat introduced from Canada (numbered B07046). Baicheng Academy of Agricultural Sciences in Jilin Province was selected. These varieties were planted in Huajialing Town, Tongwei County, Gansu Province (altitude: 2457 m; 35°22′46.3″N, 105°0′37.2″E). This location is characterized by a temperate monsoon climate, with an average annual temperature of 3.7℃ and average annual rainfall of 451.1 mm. Seeds harvested in 2020 were stored at room temperature in the forage seed room at Gansu Agricultural University in Lanzhou City. The storage room, ventilated year-round, had an average annual temperature of 10℃–12℃ and relative humidity of 45–64%.

### Seed aging treatment

Initial water content of oat seeds was measured and adjusted to 10%. According to the aging method [63], the seeds were subjected to artificial aging at 45 °C for 0, 24, 48, 72, and 96 h under 95% relative humidity. Then, the seeds were dried, restored to their original moisture content, and stored at 4 °C.

### Determination of vitality indicators

According to the method in the “Manual of Seedling Evaluation and Seed Vigor Determination Methods” [64], the oat seed germination test was conducted using petri dishes on paper, with counting completed on day 10, after which the fresh weight of seedlings was determined. Each treatment had four replications, and each replication included 50 seeds.

1 $${\rm{Germination}}\,{\rm{rate}}\,\left( {{\rm{GR}}} \right)\, = \,\left( {{{\rm{G}}_{10}}/{\rm{N}}} \right)\, \times 100\%$$

2 $${\rm{Germination}}\,{\rm{index}}\,\left( {{\rm{GI}}} \right)\,{\rm{ = }}\,\sum \,\left( {{\rm{Nt/t}}} \right)$$

3 $${\rm{Viability}}\,{\rm{index}}\,\left( {{\rm{VI}}} \right)\,{\rm{ = }}\,{\rm{GI \times S}}$$

Variables included G_10_ (number of normal seedlings on day 10), N (total seeds), Nt (number of germinated seeds per day, corresponding to t), t (days of germination), and S (fresh weight of seedlings after 10 days of germination).

### Determination of physiological and biochemical indicators

Electrical conductivity (EC) was determined by placing 50 seeds in a 150-ml triangular flask, adding 100 ml of deionized water, shaking the flask until the seeds were completely submerged, and sealing the flask with parafilm. After 24 h (± 15 min) in a 25 °C incubator, the solution conductivity was measured (H198304 [DiST4] [ Italian (HANNA]). Four replicates were used per treatment, with unseeded deionized water used as the control [65].

Malondialdehyde (MDA) content was determined using the thiobarbituric acid (TBA) method. Oat seeds (0.5 g) were homogenized with 2 ml of trichloroacetic acid (TCA), after which 3 ml of TCA was added to the homogenate before further grinding. After centrifugation at 3000 ×g for 10 min, the supernatant was collected as the extraction solution, 2 ml of which was pipetted into a stoppered test tube. Subsequently, 2 ml of TBA solution was added, the mixture was shaken before being placed in a boiling water bath. After 10 min, the solution was removed, allowed to cool, and centrifuged. Finally, optical density (OD) values were measured at 532, 600, and 450 nm. For the control, 2 ml of distilled water was used instead of the extract [65].

Soluble sugar (SS) content was determined using the anthrone method. Oat seeds (0.2 g) were added to a test tube containing 15 ml of distilled water, and the tube was placed in a boiling water bath for 20 min. The mixture was then filtered through centrifugation into a 50-ml volumetric flask. Subsequently, the residue was rinsed, constant volume, and 1 ml of the sample extract was transferred into a 20-ml graduated test tube. After adding 5 ml of anthrone reagent, the solution was vigorously mixed, and the test tube was placed in a boiling water bath for 10 min. After cooling to room temperature, the OD value at 620 nm was measured, zeroing with a blank sample [65].

Soluble protein (SP) content was determined using Thomas Brilliant Blue G-250. Oat seeds (0.5 g) were placed in a mortar, to which 5 ml of phosphate buffer (pH 7.0) was added, and the mixture was ground into a homogenate in an ice bath. Following centrifugation at 4000 ×g for 10 min, 0.1 ml of the sample extract was aspirated, to which 5 ml of Cauldron Brilliant Blue G-250 Reagent was added and mixed thoroughly. The blank control was allowed to stand for 5 min. The OD value at 595 nm was measured using a 1-cm colorimetric cup [65].

### Determination of antioxidant enzyme activities

To extract enzymes, seeds embryos (200 mg) were grounded in liquid nitrogen, homogenized in 2 ml phosphate buffer (50 mM, pH 7.0), and then centrifuged at 15 000×g for 20 min at 4℃. The supernatant was used in the antioxidant enzyme assays [66].

The activity of Superoxide dismutase (SOD) (EC 1.15. 1.1) was determined. The 3 ml reaction mixture contained 13 mM methionine, 1.3 µM riboflavin, 63 µM nitroblue tetrazolium (NBT) in 50 mM phosphate buffer (pH 7.8), and 25 µl enzyme extract. The enzyme extract was replaced with phosphate buffer in two controls. The reaction mixtures were incubated in a growth chamber (LRH-250-GII, Ningbo, China) at 25℃ under illumination. Identical tubes that were not illuminated served as blanks. After illumination for 17 min, absorbance was measured at 560 nm.

To measure Catalase (CAT) (EC 1.11.1.6) activity, 50 µl supernatant was mixed with 3.4 ml phosphate buffer (25 mM, pH 7.0, containing 0.1 mM EDTA), and 200 µl H_2_O_2_. Enzyme activity was determined by measuring the change in absorbance at 240 nm after 1 min.

### Determination of reactive oxygen species

To measure the Superoxide Anion (O_2_·^−^), Seed embryos (1 g) were grounded in liquid nitrogen, homogenized in 7 ml phosphate buffer (65 mM, pH 7.8), centrifuged at 10,000 ×g for 10 min. Then, 2 ml supernatant was mixed with 1.5 ml phosphate buffer (65 mM) and 0.5 ml hydroxylamine hydrochloride. Incubated mixture at 25℃ for 20 min, added with 2 ml sulfanilic acid (17 mM) and 2 ml α-naphthylamine (7 mM); the mixture was incubated at 30℃ for 30 min and the absorbance was measured at 530 nm [23].

To measure the Hydrogen Peroxide (H_2_O_2_) content, embryos (200 mg) were grounded and homogenized in 2.0 ml cold acetone, centrifuged at 16 000 ×g for 10 min. The supernatant (1 ml) was mixed with 100 µl 10% (w/v) titanium tetrachloride and 200 µl ammonia water, and mixed well and centrifuged at 3000 ×g for 10 min, and the supernatant was discarded. The pellet was dissolved in concentrated sulfuric acid, and then absorbance at 415 nm was recorded. A standard curve was prepared by diluting a 100 µmol/L H_2_O_2_ stock solution to 10, 20, 40, 60, 80, 100 µmol/L [23].

### Anatomical structure of oat seeds determination

The paraffin sectioning method conventionally was used. Oat seeds was soaked in distilled water for 4 h and then fixed in FAA fixative (formalin: glacial acetic acid:70% ethanol = 1:1:18) for 24 h. Gradient dehydration was performed using various concentrations of ethanol, including 30%, 50%, 70%, 85%, 95%, and anhydrous ethanol. Ethanol and xylene are transparent in steps of 45 min each (anhydrous ethanol: xylene (2:1 v/v), anhydrous ethanol: xylene (1:1), anhydrous ethanol: xylene (1:2), and xylene). The transparent material was immersed in xylene with crushed wax. The wax dipping temperature was raised from 37 °C to 56 °C. It was gradually substituted with pure paraffin wax and left in a warm box at 56 °C overnight. The wax was dipped for 2 days, with three changes of wax. After embedding, the blocks were trimmed and sliced into 10 μm thickness. The slices were baked at 45 °C. The sections were deparaffinized in xylene, rehydrated with an ethanol gradient, and double-stained with saffron-solid green. They were sealed with neutral gum, baked at 40 °C and stored permanently. The samples were observed and photographed under a microscope (Motic Panthera U). Each treatment was repeated five times, and five fields of view were selected for each sample [67].

### Ultrastructure observation of embryos and mitochondria

The transmission electron microscopy was employed. Oat seeds were soaked in distilled water for 4 h. The seed embryos were removed with a dissecting needle under a stereomicroscope, pre-fixed with 3% glutaraldehyde and then re-fixed using 1% osmium tetroxide. The embryos were dehydrated step by step in acetone (30%, 50%, 70%, 80%, 90%, 95%, 100%). Ep 812 was embedded, and the semi-thin sections were stained with toluidine blue for optical localization, and sliced on an ultrathin microtome with a diamond knife, and double-stained with uranyl acetate and lead citrate at room temperature. JEM-1400FLASH (Japan Electronics (JEOL)) transmission electron microscope was used to observe the sample sections, and photographs of cells and mitochondria were taken [68].

### Statistical analysis

Experimental data were plotted using Microsoft Excel 2010. Two-way analysis of variance (ANOVA) was performed using IBM SPSS Statistics 24.0. Adobe Photoshop CC 2018 was employed for date processing. Differences in all tests were considered statistically significant when *p* < 0.05. Data are presented as the arithmetic mean (M) ± standard error (SE).

## Data Availability

The data that support the findings of this study are available from the corresponding author upon request.

## Abbreviations

GP: germination potential
GR: germination rate
GI: germination index
VI: vigor index
GA_3_: Gibberellin
IAA: Indoleacetic acid
CTK: Cytokinins
ABA: Abscisic Acid
EC: Electrical conductivity
MDA: Malondialdehyde
TBA: thiobarbituric acid
TCA: trichloroacetic acid
OD: optical density
SS: Soluble sugar
SP: Soluble protein
SOD: Superoxide dismutase
NBT: nitroblue tetrazolium
CAT: Catalase
O_2_·^−^: Superoxide Anion
H_2_O_2_: Hydrogen Peroxide
ROS: reactive oxygen species
PCD: Programmed Cell Death
